# The feasibility of pancreatic duct stenting using a novel 4-Fr plastic stent with a 0.025-in. guidewire

**DOI:** 10.1038/s41598-021-92811-x

**Published:** 2021-07-12

**Authors:** Kazumasa Nagai, Atsushi Sofuni, Takayoshi Tsuchiya, Kentaro Ishii, Reina Tanaka, Ryosuke Tonozuka, Shuntaro Mukai, Kenjiro Yamamoto, Yukitoshi Matsunami, Yasutsugu Asai, Takashi Kurosawa, Hiroyuki Kojima, Hirohito Minami, Toshihiro Honma, Akio Katanuma, Takao Itoi

**Affiliations:** 1 Department of Gastroenterology and Hepatology, Tokyo Medical University, 6-7-1 Nishishinjuku, Shinjuku-ku, Tokyo, 160-0023 Japan; 2 Center for Gastroenterology, Teine Keijinkai Hospital, Sapporo, Japan

**Keywords:** Pancreatitis, Acute pancreatitis

## Abstract

Pancreatic duct stenting is a well-established method for reducing post-endoscopic retrograde cholangiopancreatography (ERCP) pancreatitis. However, there is no consensus on the optimal type of plastic stent. This study aimed to evaluate the feasibility and safety of a new 4-Fr plastic stent for pancreatic duct stenting. Forty-nine consecutive patients who placed the 4-Fr stent into the pancreatic duct (4Fr group) were compared with 187 consecutive patients who placed a conventional 5-Fr stent (control group). The primary outcome was technical success. Complications rate, including post-ERCP pancreatitis (PEP) were the secondary outcomes. Propensity score matching was introduced to reduce selection bias. The technical success rate was 100% in the 4Fr group and 97.9% in the control group (p = 0.315). Post-ERCP amylase level was significantly lower in the 4-Fr group than the control group before propensity score matching (p = 0.006), though without statistical significance after propensity score matching (p = 0.298). The rate of PEP in the 4Fr group (6.1%) was lower than the control group (15.5%), though without statistical significance before (p = 0.088) and after (p = 1.00) propensity score matching. Pancreatic duct stenting using a novel 4-Fr plastic stent would be at least similar or more feasible and safe compared to the conventional plastic stent.

## Introduction

Post endoscopic retrograde cholangiopancreatography (ERCP) pancreatitis is the most common complication of ERCP and can occasionally become severe or fatal. While the reported frequency of post-ERCP pancreatitis (PEP) varies between 3 and 5%, a recent systematic review reported an incidence of 14.7% in high-risk patients^1–3^. The known mechanisms of PEP comprise impaired drainage from the pancreatic duct caused by papillary edema and/or spasm of the sphincter of Oddi after the procedure^4–7^. Pancreatic duct stenting guarantees unhindered drainage of the pancreatic secretions. Moreover, it is a well-established method of reducing PEP, particularly in high-risk patients^8–15^. Endoscopic pancreatic duct stenting has also been proven effective in patients with obstructive pancreatitis^16,17^.

The size and length of the stents vary. Furthermore, there are no guidelines or consensus on an optimal plastic stent^18–20^. The use of smaller-diameter stents may result in less ductal irritation and changes, particularly in non-dilated pancreatic duct cases^18^. Despite reports on the efficacy of 3-Fr plastic stents, smaller-diameter stents have not been accepted in clinical practice^21^. This can be partially attributed to the requirement of a smaller-caliber 0.018- or 0.021-in. guidewire, which can be difficult to maneuver around the tortuous pancreatic duct compared to the standard 0.025-in. wire. This, in turn, likely results in a higher rate of PEP^6,19,21^.

Novel 4-Fr plastic stents with the ability to place over a standard 0.025-in. guidewire have been recently developed in our country. The present study aimed to examine the feasibility and safety of a new 4-Fr single-pigtail pancreatic plastic stent by comparing it with the conventional 5-Fr plastic stent.

## Results

A total of 236 patients met the eligibility criteria for study inclusion; of these, The 4-Fr plastic stent was placed 49 in patients (4-Fr group), and the conventional 5-Fr plastic stent was placed in 186 patients (conventional stent group). Table 1 summarizes the baseline characteristics, including sex, age, history of ERCP related procedures, indication for stent placement, and indication for ERCP. Indication for stent showed significant differences between groups regarding placement before propensity score matching; however, significant differences were not observed in any of these characteristics after propensity score matching.

**Table 1:** Baseline characteristics of the study patients.

Characteristics	Before propensity score matching			After propentisy score matching		
	4 Fr group (n = 49)	Conventional stent group (n = 187)	P-value	4 Fr group (n = 47)	Conventional stent group (n = 47)	P-value
Sex (M/F), n	35/14	111/76	0.122	33/14	35/12	0.818
Age, y, median (quantile)	69.0 (57.5–75.0)	67.0 (55.0–74.0)	0.521	69.0 (58.0–75.0)	68.0 (52.0–75.0)	0.555
History of ERCP related procedures, n (%)	5 (10.2)	7 (3.7)	0.135	3 (6.4)	2 (4.3)	0.646
**Indication for stent placement, n (%)**			0.028			0.928
Papillectomy	26 (53.1)	138 (73.8)		26 (55.3)	28 (59.6)	
Unintentional pancreatic guidewire passage	18 (36.7)	38 (20.3)		18 (38.3)	15 (31.9)	
Argon plasma coagulation	3 (6.1)	2 (1.1)		2 (4.3)	1 (2.1)	
Post EST bleeding	0 (0)	3 (1.6)		0 (0)	1 (2.1)	
Divisum	1 (2.0)	4 (2.1)		1 (2.1)	1 (2.1)	
Obstructive pancreatitis	1 (2.0)	2 (1.1)		0 (0)	1 (2.1)	
**Indication for ERCP, n (%)**			0.26			0.889
Ampulla of Vater adenoma	29 (59.2)	140 (74.9)		28 (59.6)	29 (61.7)	
Malignant biliary obstruction	5 (10.2)	7 (2.0)		5 (10.6)	4 (8.5)	
Biliary stone	12 (24.5)	28 (15.0)		12 (25.5)	10 (21.3)	
Benign biliary stricture	1 (2.0)	3 (1.6)		1 (2.0)	1 (2.1)	
Divisum	1 (2.0)	4 (2.1)		1 (2.1)	1 (2.1)	
Obstructive pancreatitis	1 (2.0)	2 (1.1)		0 (0)	1 (2.1)	
Post EST bleeding	0 (0)	3 (1.6)		0 (0)	1 (2.1)	

ERCP, endoscopic retrograde cholangiopancreatography; EST, endoscopic sphincterotomy.

Table 2 shows the study populations’ evaluation details, divided into endoscopic papillectomy (EP) related procedures and ERCP-related procedures other than EP. The number of patients that underwent EP and endoscopic sphincterotomy (EST) was significantly lower in 4-Fr group than conventional stent group, before (19.2% [5/26] vs 83.3% [115/138], respectively; P < 0.01) and after (19.2% [5/26] vs 82.1% [23/28], respectively; P < 0.01) propensity score matching. While, the number of patients that underwent EP and endoscopic biliary drainage (EBD) was significantly higher in 4-Fr group than conventional stent group, before (57.6% [15/26] vs 5.1% [7/138], respectively; P < 0.01) and after (57.6% [15/26] vs 14.2% [4/28], respectively; P = 0.001) propensity score matching. No significant differences were evident between the two groups for the other evaluation variables.

**Table 2:** Examination details of study populations.

Examinations		Before propensity score matching			After propentisy score matching		
		4 Fr group (n = 49)	Conventional stent group (n = 187)	P-value	4 Fr group (n = 47)	Conventional stent group (n = 47)	P-value
EP related procedure, n		26	138		26	28	
	EP alone, n (%)	5 (19.2%)	11 (8.0%)	0.139	5 (19.2%)	1 (3.6%)	0.095
	EP + EST, n (%)	5 (19.2%)	115 (83.3%)	< 0.01	5 (19.2%)	23 (82.1%)	< 0.01
	EP + EBD, n (%)	15 (57.6%)	7 (5.1%)	< 0.01	15 (57.6%)	4 (14.2%)	0.001
	EP + EST + EBD, n (%)	1 (3.8%)	5 (3.6%)	0.956	1 (3.8%)	0 (0%)	0.481
ERCP related procedure other than EP, n		23	49		21	19	
	EST, n (%)	7 (30.4%)	15 (30.6%)	0.988	7 (33.3%)	5 (26.3%)	0.629
	EST + EBD, n (%)	1 (4.3%)	5 (10.2%)	0.402	1 (4.8%)	4 (21.1%)	0.172
	Non EST + EBD, n (%)	4 (17.3%)	8 (16.3%)	0.91	4 (19.0%)	1(5.3%)	0.345
	Argon plasma coagulation, n (%)	2 (8.7%)	3 (6.1%)	0.319	2 (9.5%)	1(5.3%)	0.609
	IDUS (bile duct), n (%)	4 (17.3%)	6 (12.2%)	0.716	4 (19.0%)	3 (15.8%)	0.787
	POCS, n (%)	2 (8.7%)	2 (4.1%)	0.588	2 (9.5%)	0 (0%)	0.488

EP, endoscopic papillectomy; EST, endoscopic sphincterotomy; EBD, endoscopic biliary drainage; IDUS, intraductal ultrasonography; POCS, peroral cholangioscopy.

### Technical success and adverse events

Table 3 presents the procedure outcomes of both groups. The technical success rate was 100% (49/49) in the 4-Fr group and 98.4% (184/187) in the conventional stent group; there was no significant difference (p = 0.372). These results remained unchanged after propensity score matching (100% [47/47] vs 97.9% [46/47], respectively; P = 0.315). A significant difference between the 4-Fr group and conventional stent group was observed in stents lengths selection before (p < 0.01) and after propensity score matching (p < 0.01). The median stenting duration was 7 days in both group, and there was no difference in stenting duration between the two groups after propensity score matching (p = 0.288). However, the duration of the 4-Fr group was significantly shorter than the conventional stent group before propensity score matching(p = 0.027).

**Table 3:** Outcomes of pancreatic stent placement in each group.

	Before propensity score matching			After propentisy score matching		
	4 Fr group (n = 49)	Conventional stent group (n = 187)	P-value	4 Fr group (n = 47)	Conventional stent group (n = 47)	P-value
Technical success, no. (%)	49 (100)	184 (98.4)	0.372	47 (100%)	46 (97.9%)	0.315
**Length of PS**			< 0.01			< 0.01
3 cm, no. (%)	8 (16.3)	0 (0)		8 (17.0)	0 (0)	
5 cm, no. (%)	21 (42.9)	31 (16.8)		20 (42.6)	11 (23.9)	
7 cm, no. (%)	16 (32.7)	141 (76.2)		15 (31.9)	32 (69.6)	
9 cm, no. (%)	4 (8.2)	13 (7.0)		4 (8.5)	3 (6.5)	
**Stenting duration, days, median (quantile)**	7.0 (6.0–7.0)	7.0 (7.0–10.0)		7.0 (6.0–7.0)	7.0 (7.0–7.0)	
1–4 days, no. (%)	11 (22.9)	11 (6.0)	0.027	10 (21.7)	4 (8.7)	0.288
5–8 days, no. (%)	29 (60.4)	127 (69.0)		29 (63.0)	33 (71.7)	
9–14 days, no. (%)	3 (6.3)	20 (10.9)		3 (6.5)	4 (8.7)	
15- days, no. (%)	5 (10.4)	26 (14.1)		4 (8.7)	5 (10.9)	
**Complications during pancreatic stent placement**						
Post-ERCP pancreatitis, no. (%)	3 (6.1)	29 (15.5)	0.088	3 (6.4)	3 (6.4)	1
Hemorrhage, no. (%)	3 (6.1)	23 (12.3)	0.219	3 (6.4)	3 (6.4)	1
Spontaneous stent dislodgment, no. (%)	2 (4.1)	2 (1.1)	0.191	1 (2.1)	0 (0)	0.315
Delayed onset retroperitoneal perforation, no. (%)	0 (0)	5 (2.7)	0.587	0 (0)	0 (0)	–
Stent migration, no. (%)	0 (0)	1 (0.5)	0.608	0 (0)	1 (2.1)	0.315
**Complications after removal of pancreatic stent**						
Pancreatitis due to obstruction of pancreatic duct orifice, no. (%)	0 (0)	6 (3.2)	0.349	0 (0)	0 (0)	–

PS, plastic stent.

The PEP rate in the 4-Fr group (6.1% [3/49]) was lower than the conventional stent group (15.5% [29/187]); however, no statistical significance (p = 0.088) before propensity score matching. After propensity score matching, the PEP rate in the 4-Fr group (6.4% [3/47]) was the same as the conventional stent group (6.4% [3/47]). No significant differences were evident between the two groups regarding other complications during pancreatic stent placement. Six cases showed pancreatitis due to obstruction of pancreatic duct orifice after removing the pancreatic stent in the conventional stent group; conversely, no cases were observed in the 4-Fr group (p = 0.349) before propensity score matching.

Table 4 showed both groups’ PEP details. Post-ERCP amylase level was significantly lower in the 4-Fr group than conventional stent group before propensity score matching (165.0 vs 295.0, respectively; p = 0.006), though no statistical significance after propensity score matching (150.0 vs 240.0, respectively; p = 0.298). Asymptomatic hyperamylasemia rate was lower in the 4-Fr group (46.9% [23/49]) than the conventional group (57.2% [107/187]), though without statistical significance before (p = 0.198) and after (p = 0.536) propensity score matching. All patients with PEP recovered conservatively, including the patients with severe status in both groups.

**Table 4:** Post-ERCP pancreatitis in each group.

	Before propensity score matching			After propentisy score matching		
	4 Fr group (n = 49)	Conventional stent group (n = 187)	P-value	4 Fr group (n = 47)	Conventional stent group (n = 47)	P-value
Pre-ERCP amylase, median (quantile)	76.5 (49.8–128.8)	77.0 (62.0–107.0)	0.589	75.0 (45.3–125.8)	79.0 (56.5–100.0)	0.789
Post-ERCP amylase, median (quantile)	165.0 (110.5–369.0)	295.0 (142.5–539.0)	0.006	150.0 (106.0–383.0)	240.0 (111.0–432.0)	0.298
Asymptomatic hyperamylasemia, no. (%)	23 (46.9)	107 (57.2)	0.198	21 (44.7)	24 (51.1)	0.536
**Post-ERCP pancreatitis, no. (%)**	3 (6.1)	29 (15.5)	0.088	3 (6.4)	3 (6.4)	1
Mild, no. (%)	2 (4.1)	22 (11.7)		2 (4.3)	3 (6.4)	
Moderate, no. (%)	0 (0)	5 (2.7)		0 (0)	0 (0)	
Severe, no. (%)	1 (2.0)	2 (1.1)		1 (2.1)	0 (0)	

The supplementary table shows PEP's rate in each characteristic comparing the 4-Fr group with the conventional stent group. However, no significant differences were evident between the two groups regarding each characteristic.

## Discussion

This study demonstrated that a newly designed 4-Fr plastic pancreatic duct stent shows similar feasibility and safety compared to the conventional 5-Fr plastic stent. The new stent offered the following advantages: (1) less injury to the pancreatic duct because of small-diameter stents; (2) the ability to pass over a standard 0.025-in. guidewire, despite the small diameter; (3) easy advancement of the tapered and straight distal tip via the pancreatic duct, and (4) the role of the three flanges and a single pigtail in anchoring the stent and preventing outward and inward migration. This is the first report on the evaluation of a new 4-Fr plastic pancreatic duct stent.

Multiple clinical trials and a meta-analysis have demonstrated that pancreatic duct stenting in high-risk patients effectively reduces the incidence of PEP^8–14^. Therefore, the consensus guidelines recommend pancreatic duct stenting in high-risk patients^22,23^. However, there are controversies on the best type of plastic stent. According to previous reports, 3-or 4-Fr stents were more effective than the traditionally used 5-Fr stents. This can be attributed to their smaller diameter that causes less ductal or parenchymal pancreatic changes^18,21^. Despite the advantages above, smaller diameter stents are not widely used because of their need for a smaller caliber 0.018- or 0.021-in. guidewire. This, in turn, is more challenging to work with, thus adding to the difficulty of the procedure. Consequently, there can be a higher rate of failure of stent placement, thereby increasing the incidence of pancreatitis^24^. Despite a smaller diameter, the novel 4-Fr stents were placed into the pancreatic duct over a user-friendly standard 0.025-in. guidewire in all cases, without difficulty. The tapered and straight distal tip also enabled the easy placement of the stent in the pancreatic duct. The technical ease of stent placement is considered an important factor in selecting the prophylactic pancreatic duct stent^25^. In addition, post-ERCP amylase level and the rate of PEP in the 4-Fr stent group were lower than the conventional stent group, though without statistical significance. This 4-Fr plastic stent is expected to be at least similar or more effective for PEP prophylaxis compared to the conventional plastic stent.

Stent migration into the pancreatic duct is another adverse event associated with the procedure mentioned above. It results in stent-induced pancreatic duct changes, the need for several endoscopic attempts to retrieve the stent and an occasional surgical intervention during the failure of ERCP retrieval^26^. Inward migration did not occur in any of the cases mentioned above. The flanges and single pigtail on the proximal side of the stent might have contributed to preventing an inward migration.

The 4-Fr plastic stents have a flange at the distal end that prevents stent dislodgment. Several reports recommended the prophylactic placement of pancreatic stents without flanges. Spontaneous dislodgment occurred within 7 days in most of these cases, thus reducing the need for the re-insertion of an endoscope for stent removal^11,12,21,27^. However, early stent dislodgment may result in delayed-onset pancreatitis^14^. Delayed-onset pancreatitis because of the secondary obstruction of flow after resection or a direct burn effect in the pancreatic parenchyma is a serious complication, particularly in cases of EP^28,29^. Therefore, the inner flange is important for preventing early dislodgment, though the optimal stenting time is still uncertain^30^. In addition, stents with an inner flange were effective in cases with obstructive pancreatitis or divisum, requiring pancreatic duct drainage. Thus, the 4-Fr plastic stent with inner flange would effectively secure the pancreatic duct drainage route for the patients with a high PEP risk, including EP.

Our study had several limitations. First, it was a single-center retrospective study. Therefore, the sample size was relatively small. Second, the retrospective design might have introduced some selection bias. The study population included more patients who underwent EP than in previous studies. However, the risk of pancreatitis is high after EP^28,29,31^. The rates of PEP in our heterogeneous study population were comparable to that reported in high-risk patients with pancreatic duct stents^11,13,21^. Third, pancreatic duct stent placement was uncommon in all cases. The length of the stents and the timing of the stent removal had been selected by an operator. This might have influenced our results.

In conclusion, pancreatic duct stenting using a novel 4-Fr plastic stent is feasible and safe. Large-scale, multicenter trials are warranted to validate our results.

## Methods

### Study design

This single-center, retrospective case–control study was conducted at the Tokyo Medical University Hospital. Written informed consent was obtained from each patient before endoscopic treatment. This study was approved by the institutional review board of the Tokyo Medical University (T2020-0206), and was conducted in accordance with the ethical standards described in the latest revision of the Declaration of Helsinki. Informed consent for patient participation was received in the form of an opt-out in-hospital notice.

### Study participants

A total of 895 consecutive ERCP procedures were performed between November 2019 and March 2021. We selected 49 consecutive patients who had a 4-Fr plastic stent placed in their pancreatic duct. We also included 187 consecutive patients who had a conventional 5-Fr pancreatic duct stent with internal flanges (Geenen, Pancreatic Stent Sets, Cook Medical, Bloomington, IN, USA) in their pancreatic duct between November 2013 and October 2019 as the control group. The study primarily included the following patients who were considered at a high risk of developing PEP: (1) those receiving unintentional pancreatic guidewire passages, (2) requiring EP, (3) requiring argon plasma coagulation (APC) for residual lesions of the papilla tumor after EP, (4) those showing post-EST bleeding. The remaining patients were placed on a stent for pancreatic duct drainage in divisum or obstructive pancreatitis. The exclusion criteria were as follows: (1) age < 18 years and (2) refusal to participate in the study. A history of biliary or pancreatic drainage during patient selection was not considered. We compared the 4-Fr group and control group with respect to outcomes. Propensity score matching was introduced to reduce selection bias.

### Endoscopic procedures

**Figure 1:**
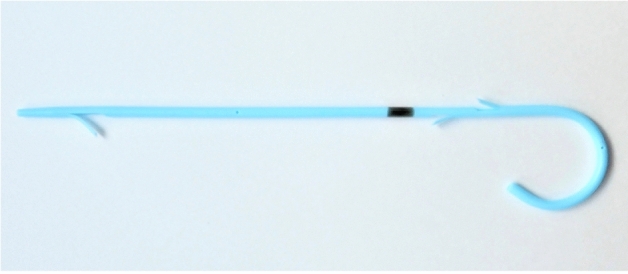
4-Fr pancreatic stent. The stent has a tapered tip, three internal flanges (one at the distal end and two at the proximal end), and a single external pigtail with a black marker on the proximal side.

All patients underwent ERCP using a duodenoscope (TJF-260V; Olympus Medical Systems, Tokyo, Japan) under conscious sedation. Cannulation was attempted using a standard injection catheter (ERCP catheter, MTW Co., Dusseldorf, Germany) or sphincterotome (CleverCut; Olympus Medical Systems, Tokyo, Japan) with a 0.025-in. guidewire (VisiGlide 2, Olympus Medical Systems, Tokyo, Japan). We used a newly designed 4-Fr pancreatic duct stent with varying lengths of 3, 5, 7, and 9 cm. The stent had a tapered tip, three internal flanges (one at the distal end and two at the proximal end), and a single external pigtail (Fit Stent 025: Gadelius Medical Co., Ltd., Tokyo, Japan) (Fig. 1). The stent could pass over a 0.025-in. guidewire under fluoroscopic guidance.

**Figure 2:**
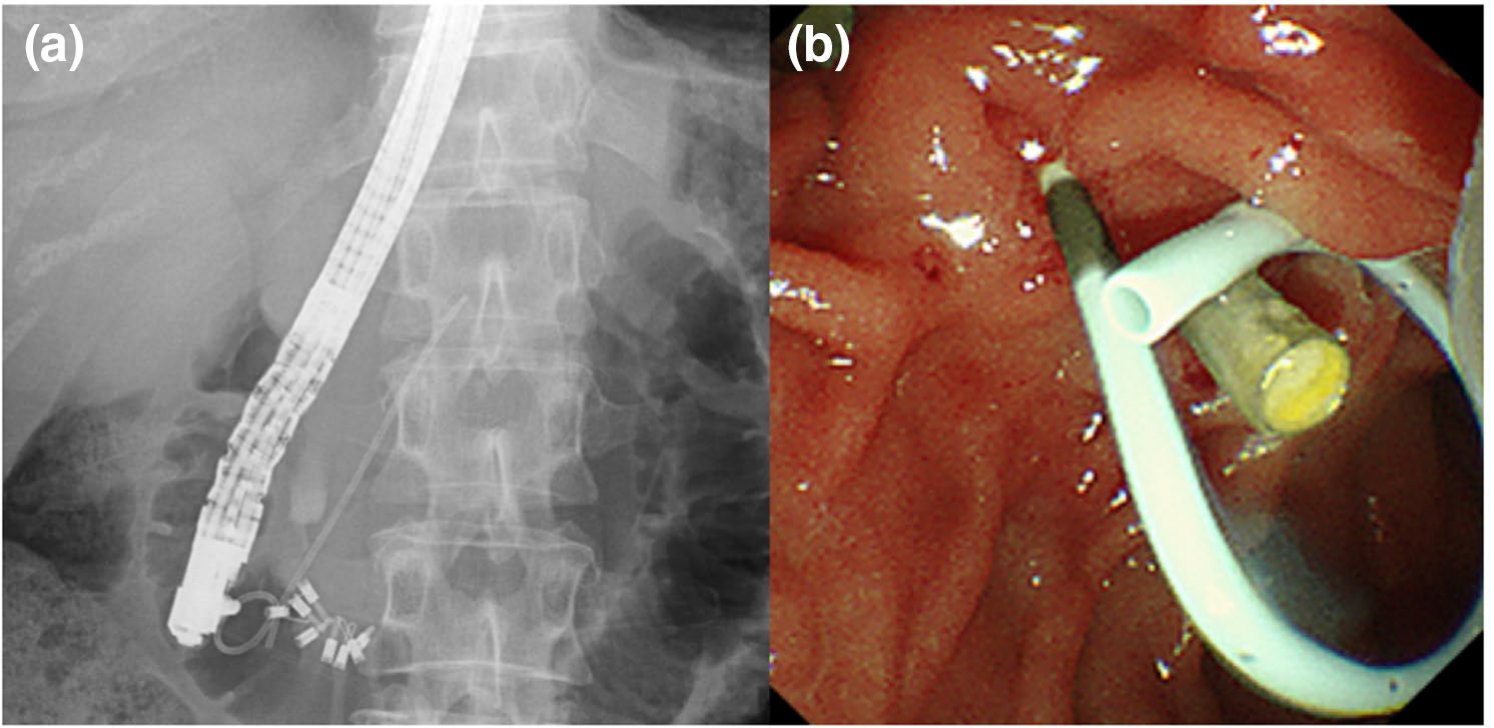
A case of pancreatic duct stenting using the novel 4-Fr plastic stent. (**a**) Fluoroscopy, The 4-Fr stent has been placed in the pancreatic duct; (**b**) Endoscopy, The single-pigtail and the black marker of the proximal side are exposed to the duodenum.

The patients underwent subsequent pancreatic duct cannulation, contrast injection, and guidewire insertion for stent placement. We selected the length of the stent based on the degree of flexion and the length of the pancreatic duct in the head of the pancreas. The stent was passed over a guidewire under fluoroscopic guidance (Fig. 2). Following ERCP, the patients were requested to fast until their blood tests confirmed no pancreatitis or other complications, the following day. All patients were hospitalized for ERCP and observation. The stent was planned to be removed duodenoscopically between the third to seventh day in cases of prophylactic pancreatic stent placement, if it had not been dislodged. However, it was planned to be removed based on the symptoms in case of pancreatic duct drainage. The conventional 5-Fr pancreatic duct stent placement was the same as those for 4-Fr pancreatic duct stent. All ERCP procedures were performed by experts (> 5 years of ERCP experience) or by trainees (< 5 years of ERCP experience) under the direction of an expert.

### Measured outcomes

The primary outcome was technical success, defined as the successful placement of the stent. The secondary outcomes comprised the frequency and severity of PEP, hyperamylasemia, rate and duration of spontaneous stent dislodgment, stent migration, and other complications. We also evaluated the PEP rate and analyzed various risk factor;. 4-Fr group and control group were compared regarding these outcomes. All ERCP-related complications were graded according to the severity grading system of the American Society for Gastrointestinal Endoscopy Lexicon^32^. PEP was defined as pancreatic pain and hyperamylasemia within 24 h of the procedure. Hyperamylasemia was defined as an increase in the serum amylase level to more than thrice the upper normal limit. We defined “stent migration” as the inward migration of the stent into the pancreatic duct. We defined “stent dislodgment” as the outward migration of the stent to the duodenal side.

### Statistical analyses

Categorical variables were expressed as numbers and percentages, and they were compared using the χ^2^ test (with Yates’ correction) or the Fisher’s exact test. Continuous variables were expressed as medians and interquartile ranges, and they were compared using the Mann–Whitney U-test. We used propensity score matching to adjust baseline differences between the two groups. Sex, age, history of ERCP related procedures, indication for pancreatic stent placement, and indication for ERCP were selected as the observed covariates. Based on this set of covariates, propensity scores were estimated using a logistic regression model. Groups were matched using 1:1 nearest neighbor-matching, within a caliper width of 0.2 of the standard deviation of the propensity score logit. After propensity matching, differences in clinical outcomes were compared between the two groups. All analyses were performed using IBM SPSS software (version 27; IBM Corp., Armonk, NY, USA). For all analyses, P < 0.05 was considered statistically significant.

### Ethical statement

This study was approved by the institutional review board of the Tokyo Medical University (T2020-0206), and was conducted in accordance with the ethical standards described in the latest revision of the Declaration of Helsinki.

### Patient consent for patient participation and publication

Informed consent for patient participation was received in the form of an opt-out in-hospital notice.

## Supplementary Information

Supplementary Information.
