# Supplementary material for: The feasibility of pancreatic duct stenting using a novel 4-Fr plastic stent with a 0.025-in. guidewire
Source: Sci Rep. 2021 Jul 12;11:14285. doi: 10.1038/s41598-021-92811-x (PMC8275660; doi:10.1038/s41598-021-92811-x)
Supplement: Supplementary file 1 — Supplementary Information. [file 41598_2021_92811_MOESM1_ESM.docx]

| Supplementary table. The rate of post-ERCP pancreatitis in each characteristic | | |  |  |  |  |  |
| --- | --- | --- | --- | --- | --- | --- | --- |
|  | Post-ERCP pancreatitis -Before propensity score matching- | | |  | Post-ERCP pancreatitis -After propensity score matching- | | |
|  | 4 Fr group (%) | Conventional stent group (%) | P-value |  | 4 Fr group (%) | Conventional stent group (%) | P-value |
| Sex |  |  |  |  |  |  |  |
| Male | 3/35 (8.6) | 11/111 (9.9) | 0.815 |  | 3/33 (9.1) | 2/35 (5.7) | 0.668 |
| Female | 0/14 (0) | 18/76 (23.7) | 0.064 |  | 0/14 (0) | 1/12 (8.3) | 0.462 |
| Age, y |  |  |  |  |  |  |  |
| ≦60 | 0/16 (0) | 14/71 (19.7) | 0.052 |  | 0/14 (0) | 2/16 (12.5) | 0.485 |
| >60 | 3/33 (9.1) | 15/116 (12.9) | 0.764 |  | 3/33 (9.1) | 1/31 (3.2) | 0.614 |
| History of ERCP related procedures | 0/5 (0) | 1/7 (14.3) | 0.377 |  | 0/3 (0) | 1/2 (50) | 0.4 |
|  |  |  |  |  |  |  |  |
| Indication for stent placement |  |  |  |  |  |  |  |
| Papillectomy | 3/26(11.5) | 22/138 (15.9) | 0.567 |  | 3/26(11.5) | 2/28 (7.1) | 0.663 |
| Unintentional pancreatic guidewire passage | 0/18 (0) | 5/38 (13.2) | 0.164 |  | 0/18 (0) | 0/15 (0) | - |
| Argon plasma coagulation | 0/3 (0) | 0/2 (0) | - |  | 0/2 (0) | 0/1 (0) | - |
| Post EST bleeding | 0/0 (0) | 1/3 (33.3) | - |  | 0/0 (0) | 1/1 (100) | - |
| Divisum | 0/1 (0) | 1/4 (25.0) | 0.576 |  | 0/1 (0) | 0/1 (0) | - |
| Obstructive pancreatitis | 0/1 (0) | 0/2 (0) | - |  | 0/0 (0) | 0/1 (0) | - |
|  |  |  |  |  |  |  |  |
| Indication for ERCP |  |  |  |  |  |  |  |
| Ampulla of Vater adenoma | 3/29 (10.3) | 22/140 (15.7) | 0.459 |  | 3/28 (10.7) | 2/29 (6.9) | 0.67 |
| Malignant biliary obstruction | 0/5 (0) | 1/7 (14.3) | 0.377 |  | 0/5 (0) | 0/4 (0) | - |
| Biliary stone | 0/12 (0) | 4/28 (14.3) | 0.297 |  | 0/12 (0) | 0/10 (0) | - |
| Benign biliary stricture | 0/1 (0) | 0/3 (0) | - |  | 0/1 (0) | 0/1 (0) | - |
| Divisum | 0/1 (0) | 1/4 (25.0) | 0.576 |  | 0/1 (0) | 0/1 (0) | - |
| Obstructive pancreatitis | 0/1 (0) | 0/2 (0) | - |  | 0/0 (0) | 0/1 (0) | - |
| Post EST bleeding | 0/0 (0) | 1/3 (33.3) | - |  | 0/0 (0) | 1/1 (100) | - |
|  |  |  |  |  |  |  |  |
| EP related procedure |  |  |  |  |  |  |  |
| EP alone | 0/5 (0) | 2/11 (18.2) | 0.308 |  | 0/5 (0) | 0/1 (0) | - |
| EP+EST | 1/5 (20.0) | 19/115 (16.5) | 0.838 |  | 1/5 (20.0) | 2/23 (8.7) | 0.459 |
| EP+EBD | 2/15 (13.3) | 1/7 (14.3) | 0.952 |  | 2/15 (13.3) | 0/4 (0) | 0.44 |
| EP+EST+EBD | 0/1 (0) | 0/5 (0) | - |  | 0/1 (0) | 0/0 (0) | - |
|  |  |  |  |  |  |  |  |
| ERCP related procedure other than EP |  |  |  |  |  |  |  |
| EST | 0/7 (0) | 3/15 (20.0) | 0.523 |  | 0/7 (0) | 0/5 (0) | - |
| EST+EBD | 0/1 (0) | 0/5 (0) | - |  | 0/1 (0) | 0/4 (0) | - |
| non EST+EBD | 0/4 (0) | 0/8 (0) | - |  | 0/4 (0) | 0/1 (0) | - |
|  |  |  |  |  |  |  |  |
| Argon plasma coagulation | 0/2 (0) | 0/3 (0) | - |  | 0/2 (0) | 0/1 (0) | - |
| IDUS (bile duct) | 0/4 (0) | 1/6 (16.7) | 0.389 |  | 0/4 (0) | 0/3 (0) | - |
| POCS | 0/2 (0) | 0/2 (0) | - |  | 0/2 (0) | 0/0 (0) | - |
| EP, endoscopic papillectomy; EST, endoscopic sphincterotomy; EBD, endoscopic biliary drainage; IDUS, intraductal ultrasonography; POCS, peroral cholangioscopy. | | | | | | | |
